# Practical adjustment of blood hemoglobin for anemia treatment in maintenance hemodialysis

**DOI:** 10.1093/ckj/sfaf274

**Published:** 2025-09-05

**Authors:** Masayuki Tanemoto

**Affiliations:** Division of Nephrology, Department of Internal Medicine, Tokatsu Clinic, Chiba, Japan

To the Editor,

In patients undergoing maintenance hemodialysis, situations during blood sampling, such as patient posture and interdialytic weight gain (IDWG), affect the measured value of pre-dialysis blood hemoglobin concentration (preHb). Plasma redistribution due to seated-to-supine postural change dilutes blood and reportedly lowers preHb by ∼5% [1]. Additionally, while one report not accounting for IDWG shows only a 0.9% difference in preHb between midweek (i.e. Wednesday/Thursday) and early-in-the-week (i.e. Monday/Tuesday) dialysis sessions, other reports accounting for IDWG indicate that every 1% increase in IDWG relative to dry weight (DW, the target weight after each dialysis session) lowers preHb by ∼3% [2, 3]. The combined effects of postural change and IDWG can result in significant preHb variation, as observed in a patient who had postural change and wide variation in IDWG/DW (from 0.005 to 0.065) at routine blood sampling. Depending on blood sampling situations, this patient presented preHb varying from 9.5 to 12.5 g/dl.

Whereas the Kidney Disease: Improving Global Outcomes and Kidney Disease Outcomes Quality Initiative guidelines do not specify blood sampling situations, they are based on studies conducted in Western countries, where midweek pre-session seated sampling is the standard [4]. Therefore, in anemia management, which is recommended on Hb levels, it is necessary to adjust Hb or its target levels according to specific blood sampling situations. However, even after subsequent controversy conferences [5], these guidelines present a “one-size-fits-all” approach for anemia management.

For supine sampling, Hb should be increased by 5% (∼0.5 g/dl). Additionally, since the average midweek pre-session IDWG/DW is ∼3% [4], Hb should be increased by 3% (∼0.3 g/dl) for every 1% excess in IDWG/DW >3%, and decreased by 3% for every 1% shortage in IDWG/DW <3%. Table 1 summarizes the adjustments according to blood sampling situations and presents calculation formulas for adjustment. Without such modification, anemia management following the guidelines may lead to over or under correction. Notably, patients who experience large IDWG may have higher-than-recommended Hb levels. For instance, in the aforementioned case, where preHb in the guideline-presumed blood sampling situations (seated sampling under IDWG/DW 0.03) is calculated to be ∼11 g/dl, if preHb 9.5 g/dl in supine sampling under IDWG/DW 0.065 is managed to 11.5 g/dl, this management will make preHb >13 g/dl, the levels not recommended, in the guideline-presumed situations. Such management may elevate thrombotic risks.

**Table 1:** Adjustment of preHb according to blood sampling situations.

Situation	Adjustment
Patient posture	
Seated	Unnecessary
Supine	Increase by 5% (∼0.5 g/dl)
IDWG/DW	
>3%	Increase by 3% (∼0.3 g/dl) for every 1% excess
<3%	Decrease by 3% (∼0.3 g/dl) for every 1% shortage
	Formula
Seated	preHb + 3× (preBW/DW − 1.03) × preHb
	(approximately) preHb + 30× (preBW/DW − 1.03)
Supine	preHb + 3× (preBW/DW − 1.03) × preHb + 0.05× preHb
	(approximately) preHb + 30× (preBW/DW − 1.03) + 0.5

IDWG, interdialytic weight gain; DW, dry weight; preHb, pre–dialysis blood hemoglobin concentration in g/dl; preBW, pre-dialysis body weight.

For example, preHb 11 g/dl at preBW 71.5 kg of a 68-kg-DW patient needs to be adjusted as follows.

For seated sampling,

adjusted preHb = 11 + 3× (71.5/68 − 1.03) × 11 = 11.7

or (approximately) = 11 + 30× (71.5/68 − 1.03) = 11.6.

For supine sampling,

adjusted preHb = 11 + 3× (71.5/68 − 1.03) × 11 + 0.05 × 11 = 12.3

or (approximately) = 11 + 30× (71.5/68 − 1.03) + 0.5 = 12.1.
